# JAK2 and TET2 Mutation in Polycythemia Vera

**DOI:** 10.7759/cureus.17854

**Published:** 2021-09-09

**Authors:** Jaskamal Padda, Khizer Khalid, Jayant Yadav, Abdulelah H Almanie, Krutagni Adwait Mehta, Hussam Al Hennawi, Nymisha L Boddeti, Victor Yosef Melt Campos, Gutteridge Jean-Charles

**Affiliations:** 1 Internal Medicine, JC Medical Center, Orlando, USA; 2 Internal Medicine, Advent Health & Orlando Health Hospital, Orlando, USA

**Keywords:** tet2, jak2, polycythemia vera, stat5, myeloproliferative neoplasms

## Abstract

The *Ten-Eleven Translocation-2* (*TET2*) gene, located on chromosome 4q24, has been implicated in hematological malignancies. The *TET2* gene shows mutations in variable myeloid malignancies with the involvement of 15% of myeloproliferative neoplasms (MPNs). The inactivation of the *TET2* gene in both mice and humans has shown a high degree of deregulation of the hematopoiesis process leading to hematological malignancies. Polycythemia vera (PV), an MPN characterized by increased red blood cell mass, has been associated with the *TET2* gene. Furthermore, *TET2* genes have been found to facilitate *Janus kinase-2* and signal transducer activator of transcription 5, as well as modulate the epigenetic composition of genomic DNA. However, little is known about the role of *TET2* mutations in patients with PV. Several studies have been conducted to further assess the significant role of *TET2 *gene function in various disease processes and prognoses to enhance the management and care of these patients.

## Introduction and background

Myeloproliferative neoplasms (MPNs) include polycythemia vera (PV), essential thrombocythemia (ET), primary myelofibrosis (MF), and chronic myeloid leukemia (CML) among several other disorders. CML is a Philadelphia chromosome-positive MPN, whereas others are negative for the Philadelphia chromosome. Several genes are implicated in myeloproliferative diseases including PV. In 2005, a recurrent point mutation in *Janus kinase-2* (*JAK2*) exon 14 was identified in patients with MPNs. This mutation results in valine-to-phenylalanine substitution at position 617 in JAK homolog autoinhibitory domain. This substitution results in a gain-of-function of *JAK2* which autonomously activates downstream signaling pathways, including Janus kinase/signal transducer and activator of transcription (JAK-STAT), phosphatidylinositol 3-kinase/protein kinase B, and extracellular signal-regulated kinases/microtubule-associated protein kinase [1,2]. Mutations in *JAK2* exon 12 and *MPL515* genes have also been implicated in MPNs which alter the JAK-STAT signaling pathway. However, the contribution of these genes has not been clearly identified in MPN phenotype. Myeloproliferative leukemia (MPL) and *JAK2*, although strongly associated with MPN, do not necessarily specify clinicopathologic correlation. Growing evidence suggests the role of other genetic factors impacting the pathogenesis of MPNs. PV shows a higher expression of *JAK2*, but at the same time, *JAK2* is not exclusive to PV in the MPN spectrum. Similar data have also been seen with the MPL mutant gene. This signifies the importance of the identification of more molecular alterations [1-5]. In recent studies, along with JAK2V617F, a coexisting *Ten-Eleven Translocation-2* (*TET2*) gene, loss-of-function mutation has been found in the minimal loss of heterozygosity region of chromosome 4q24 in MPN patients [2]. *TET2* has pleiotropic roles during hematopoiesis, including stem-cell self-renewal, lineage commitment, and terminal differentiation of monocytes [6]. *TET2* is a known tumor-suppressor gene.

PV is a monoclonal proliferative disorder of multipotent myeloid progenitor cells, increasing the cell count of all three lineages [7]. The disorder has a prevalence of 0.68-2.6 per 100,000 individuals [8]. It is characterized by increased red blood cell (RBC) mass and is associated with an increased risk of thrombotic events, leukemic transformation, and MF. JAK2V617F mutations are found in greater than 95% of PV patients. However, in patients with PV who were negative for JAK2V617F, other abnormalities were found in *JAK2* exon 12 which induced activation of the JAK-STAT pathway at a greater level than the JAK2V617F allele [3]. This has been the basis for the World Health Organization’s definition of PV or other myeloproliferative disorders including ET which was revised in 2008. The diagnostic criteria include the detection of *JAK2* mutation in exon 12 or 14. However, it is unclear how a single mutation in *JAK2* can lead to different clinical phenotypes of MPN. *JAK2* mutations do not explain the variable prognosis among patients with PV, which can perhaps be explained by the variable burden of this mutation in hematopoietic cells as well in genes other than *JAK2*. Furthermore, variations in the expression of alternative genes and epigenetic modification may account for some of these disparities. Several other genes are implicated in PV including *TET2* genes [4]. *TET2* genes facilitate *JAK2* or STAT5 signal transduction as well as modulate the epigenetic composition of genomic DNA, including DNA and histone methylation and acetylation [5].

*TET2* mutations often occur early during the development of human myeloid malignancies, including PV, ET, MF, myelodysplastic syndrome (MDS), chronic myelomonocytic leukemia (CMML), and acute myeloid leukemia (AML). These mutations appear to target hematopoietic stem/progenitor cells [7,8]. However, studies have shown that *TET2* gene mutations may also occur during later stages, which may explain the transformation of MPN to acute leukemia [6]. This implies that therapeutic targets may have to focus on hematopoietic stem cells or progenitor cells to eradicate these myeloproliferative conditions. Table 1 illustrates *TET2* mutation prevalence in different myeloid malignancies [9], whereas Table 2 demonstrates the prevalence of *TET2* mutation in MPNs [10].

**Table 1:** Prevalence of TET2 mutation in myeloid malignancies [9]. *TET2*: *Ten-Eleven Translocation-2*

Myeloid malignancies	TET2 mutation prevalence
Acute myeloid leukemia	12–24%
Chronic myelomonocytic leukemia	20–40%
Myelodysplastic syndromes	19–26%
Myeloproliferative neoplasms	7–13%
Systemic mastocytosis	29%

**Table 2:** Prevalence of TET2 mutation in myeloproliferative neoplasms [10]. *TET2*: *Ten-Eleven Translocation-2*

Myeloproliferative neoplasms	TET2 mutation prevalence
Polycythemia vera	16.8%
Essential thrombocythemia	9.8%
Myelofibrosis	15.7%

## Review

Overview of *JAK2* and *TET2* gene

**Figure 1:**
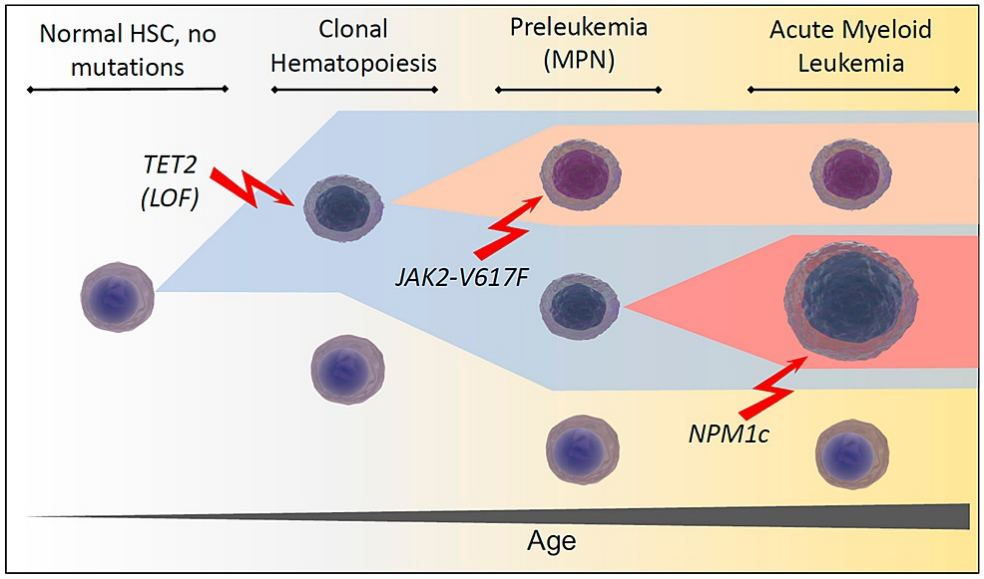
The progression of HSC to AML - TET2 and JAK2. LOF: loss of function; HSC: hematopoietic stem cells; MPN: myeloproliferative neoplasm; JAK2V617F: Janus kinase 2; NPM1c: nucleophosmin; *TET2*: *Ten-Eleven Translocation-2*; AML: acute myeloid leukemia Copyright/License: Licensee MDPI, Basel, Switzerland. This figure is from an open-access article distributed under the terms and conditions of the Creative Commons Attribution (CC BY) license (http://creativecommons.org/licenses/by/4.0/). No modifications were made to the original figure. Perner F, Perner C, Ernst T, Heidel FH: Roles of JAK2 in aging, inflammation, hematopoiesis and malignant transformation. Cells. 2019, 8:854. 10.3390/cells8080854 [15].

In 2009, the *TET2* gene was described in myeloid malignancies along with its variants. The *TET2* gene is located on chromosome 4q24. *TET2* protein modulates DNA hydroxymethylation through the conversion of 5-methylcytosine to 5-hydroxymethylcytosine, promoting DNA demethylation. The functional domain of *TET2* is located at the C-terminus; it consists of a cysteine-rich domain as well as a double-stranded β-helix fold domain. Significant functions of *TET2* include the hematopoiesis role, stem-cell self-renewal promotion, monocyte differentiation on the terminal stage, and lineage commitment. In addition, the *TET2* gene is highly expressed in hematopoietic progenitor cells [11]. *TET2* gene shows mutations in variable myeloid malignancies with the involvement of 15% of the MPNs. The inactivation of the *TET2* gene in both mice and humans has shown a high degree of deregulation of the hematopoiesis process leading to hematological malignancies [12]. Moreover, the *TET2* gene has been described as a tumor-suppressor gene with its homozygous and heterozygous mutations leading to hematopoietic malignancies in humans. Amino acid substitutions, frameshifting, in-frame deletions, and generated stop codons are all possible *TET2* gene mutations. However, there is no precise pattern of genotypes with the associated hematological malignancies such as MDS, CMML, and AML [11]. Jung-Sook et al. reported that all patients carrying the *TET2* mutation also carried the JAK2V617F mutation. There was no relation between the occurrence of the *TET2* mutation and age, JAK2V617F allele burden, frequency of organomegaly, fibrosis of the marrow, hematologic indices, as well as thrombotic or hemorrhagic complications in all MPN patients [13]. Most *TET2* mutations result in the loss of enzymatic function. The most common types of mutations are nonsense and frameshift ones, which occur before the C-domain. However, missense mutations and in-frame deletions also occur within the C-domain [14]. Figure 1 demonstrates the loss of function of *TET2* along with mutation of JAK2V617F, leading to MPN and AML [15].

Clinical implications of *TET2* gene mutation and its association with polycythemia (in terms of diagnosis, treatment targets, and prognosis)

Patients with PV who are homozygous for JAK2V617F are prone to developing post-PV complications such as MF [16,17]. In addition, patients with JAK2V617F mutation who acquire BCR-ABL1 translocation are prone to developing CML. However, the role of *TET2* in the progression or evolution of PV to MF, AML, or CML has not been established. Much controversy has been elicited regarding the prognosis of myeloid malignancies with *TET2* mutations [18]. According to Tefferi et al., there were no clinically significant alterations in the prognosis or overall survival rate in patients diagnosed with MPNs based on findings from large cohort studies [19]. On the other hand, studies have demonstrated poorer outcomes in hematologic malignancies including CMML and AML associated with *TET2* mutations with limited data on PV [20,21]. Moreover, *TET2* mutations associated with malignancies have been shown to have a favorable response to hypomethylating agents in high-risk patients [11,22].

Uncertain prognosis in patients with *TET2* mutations is largely due to studies lacking evidence of possible underlying and other associated mutations for the prognosis of *TET2* mutations. In other words, there is a lack of evidence regarding whether *TET2* mutations primarily dysregulate well-known pathways associated with hematopoietic transformation or constitute a novel, poorly discovered pathway toward malignancy pathogenesis [21]. To further elaborate, *TET2* mutations may set the stage toward the pathogenesis of different hematologic malignancies and act jointly with other gene mutations at early stages. For example, *TET2* mutations when combined with *JAK2* and *ASXL1* mutations give rise to PV and MF [23]. Overall, different combinations of gene mutations along with *TET2* mutations markedly reflect on the prognosis. Of note, Swierczek et al. have suggested that *TET2* mutations are not the PV initiating cascade, rather they occur after *JAK2* mutations. It has also been suggested that combined *JAK2* and *TET2* mutations allow for dramatic proliferation over *TET2*-negative PV subclones, emphasizing the hypothesis that *TET2* mutations increase the aggressive nature of *JAK2* mutation-positive PV [24]. Interestingly, Ortmann et al. found that the order in which *JAK2/TET2* mutations are acquired reflects on the clinical prognosis of patients diagnosed with PV. Patients with initial *JAK2* mutation demonstrated a higher risk of thrombosis compared to a more indolent course in patients with initial PV-associated *TET2* mutation [25].

With regard to the treatment, studies have shown that management with peginterferon alfa-2a can reduce JAK2V617F clones but not the *TET2* mutant ones. As mentioned earlier, *TET2* mutation leads to persistent clonal hematopoiesis [26]. Another study has shown that peginterferon alfa-2a-treated patients with both *JAK2* and *TET2* mutations had a less significant reduction in the burden of JAK2V617F compared to those with *JAK2* but without *TET2* mutations. The former group possessed a higher burden of JAK2V617F mutation at the beginning of the therapy. Furthermore, the same study revealed that patients without complete remission were more likely to have additional mutations apart from *JAK2* [27].

## Conclusions

Several studies suggest that *TET2* mutations are an early event in the development of myeloid malignancies, yet their function in normal cells and pathologic conditions remains to be elucidated. It is unclear what triggers these mutations and at what point in time. *JAK2* mutation is associated with most MPNs including PV. However, a single mutation in *JAK2* does not explain the variable prognoses among patients with PV. Several other genes including mutations in *TET2* have been implicated. *TET2* genes facilitate *JAK2* or STAT5 signal transduction and allow the accelerated proliferation of cells in patients with *JAK2*-positive PV. Further, studies have shown that patients with initial *JAK2* mutation demonstrated a higher risk of thrombosis compared to a more indolent course in patients with initial PV-associated *TET2* mutation. However, these are early days and much remains unknown about *TET2* mutations. As newer studies continue to shed light on this subject, whether *TET2* genes may be targeted as a part of disease management or prognostication remains to be seen. Targeted disruption of genes in animal models, as well as perturbation of *TET2* levels in normal and malignant cell types in vitro and in vivo, may offer clues to the understanding of the function of *TET2*.
